# Primary Malignant Melanoma of the Uterine Cervix Treated with Ultraradical Surgery: A Case Report

**DOI:** 10.5402/2011/683020

**Published:** 2010-10-05

**Authors:** Luz Calderón-Salazar, David Cantú de Leon, Delia Perez Montiel, Erika Almogabar-Villagrán, Verónica Villavicencio, Lucely Cetina

**Affiliations:** ^1^Department of Gynecologic Oncology, Instituto Nacional de Cancerologia, 14080 Mèxico, Mexico; ^2^Department of Pathology, Instituto Nacional de Cancerologia, 14080 Mèxico, Mexico; ^3^Department of Surgery, Instituto Nacional de Cancerologia, 14080 Mèxico, Mexico; ^4^Department of Medical Oncology, Instituto Nacional de Cancerologia, 14080 Mèxico, Mexico

## Abstract

Primary melanomas of the uterine cervix are rare tumors with no more than 60 cases reported in the world literature. Poor prognosis is considered for the neoplasia itself as well as for diagnostic tardiness. There is no standard treatment; however, radical surgery is the treatment cornerstone. Our aim was to present the case of a 34-year-old woman with a primary malignant melanoma in the uterine cervix with affectation of the posterior face of the vagina without metastasis. Total infraelevator pelvic exenteration and adjuvant radiotherapy was performed. The patient was under surveillance for 8 years of followup without evidence of local or distant disease. The majority of case reports found suggests radical hysterectomy as the treatment indicated for these patients. Notwithstanding this, survival is very short when patients are treated in this manner. Based on our results and on those reported in the literature, we propose initial treatment with total pelvic exenteration as optimal management for this neoplasia in its initial form.

## 1. Introduction

Primary malignant melanoma of the cervix constitutes a rare disease [1–4], representing less than 2% of cases of malignant melanoma that affects the female genital tract [1]. It was not until 1960, after the description by Cid [5] of melanocytic cells in the cervix, when the concept was accepted of primary melanoma in uterine cervix. It is considered as a very aggressive neoplasia [6], whose diagnosis is untimely due to the unusualness of the disease and the scarce suspicion of the clinician. 

At present, there is no consensus on the therapeutic approach to follow [1, 2, 7]; in the reports found, initial treatment is referred of radical hysterectomy accompanied or not by radical pelvic lymphadectomy or superior vaginectomy [1, 2, 6–11]. 

Although there are no significant statistics, prognosis in general is poor [2, 9, 11] and unpredictable [1] and is even worse when early visceral metastases are discovered [11]. In the current literature, there are no more than 60 cases reported of this entity.

Our purpose is to report the case of a patient with this neoplasia who was treated with ultraradical surgery followed by radiotherapy and who has had long-term followup.

## 2. Case Report

A female patient 34 years of age, Mexican mestizo, nubile. History of 2 months of abnormal genital bleeding, for which she was evaluated by abdominal Ultrasound (US) with a report of uterine myomatosis. Carrying out a gynecological examination is decided upon, in which a blackish lesion is observed in the area of the uterine cervix. Thus, a biopsy of the cervix was taken, which revealed melanoma. The patient was therefore referred to the National Cancerology Institute (INCan) in Mexico City in January 2001.

**Figure 1:**
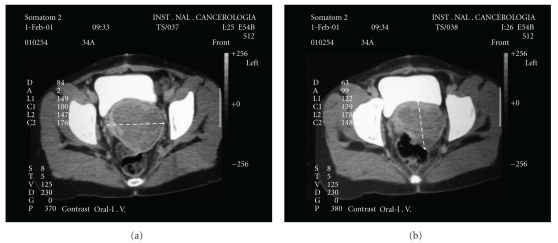
Contrast enhanced CT scan of the pelvis showing a solid cervical mass without evidence of direct involvement of bladder or rectum.

On initial physical exploration at this Institute, cervix-dependent tumor was noted that extended to the right pelvic wall, of petraeus consistency, and another at the level of the anterior wall of the vagina. A new biopsy was taken, reporting malignant melanoma. Chest X-ray, cystoscopy, and colonoscopy were performed, and reported as normal, in addition to complete revision of skin and eyes, discarding other sites of melanocytic lesions. Additionally, a Computed tomography (CT) was requested, which reported tumor in the pelvic floor toward the right annex of approximately 8 × 6 cm, which displaced the bladder and ureters (Figure 1). Due to the extension of the lesion, the patient was considered to be a candidate for exploratory laparotomy, and total pelvic exenteration was planned, which was performed in March 2001. Reconstruction of the digestive and urinary tracts was carried out with a terminal colostomy and Bricker ileal conduit operation, respectively. The final histopathological report of the surgical specimen obtained demonstrated malignant melanoma of the cervix of 7.5 × 5 cm, surgical margins were reported as negative. Intrahospital evolution was satisfactory, and the patient was discharged on day 10. Adjuvant radiotherapy was administered, 21 Gy in three sessions, concluding in June 2001. The patient has been under routine surveillance for 8 years, and has only referred four occasional urinary tract infections, which have been resolved medically. To the moment of this report, there has been no evidence of local or distant recurring disease.

## 3. Pathology

**Figure 2:**
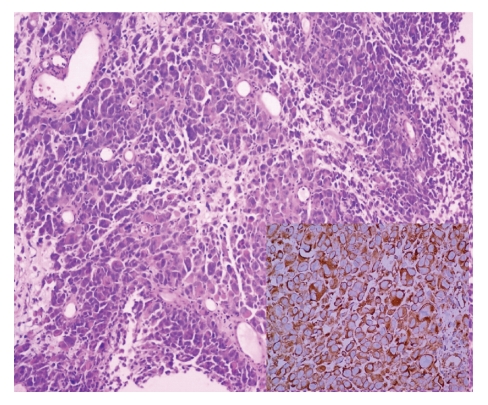
Microscopic picture showing a medium power view of the lesion. (HE 10x) in the box Melan A reaction diffuse positive.

For histopathological studies, the product of the total pelvic exenteration was received, in which a neoplastic lesion of the cervix measured 7.5 × 5.5 cm with involvement of the upper third of the vagina into anterior vaginal septum. The neoplasia was polypoid and light brown in color with necrosis. Microscopically, the neoplasia was formed by nodules of broad cytoplasmic cells with poorly defined borders with pleomorphic nuclei with prominent nucleolae. These areas displayed transition with areas with fascicules of elongated cells of moderate cytoplasm and similar elongated nuclei to those described previously (Figure 2). 

Immunohistochemical stains were positive for S-100 protein, Melan A, and HMB 45 in neoplastic cells. CKAE1/AE3 and CEA were negative. No binding activity was found. A total of 15 pelvic lymph nodes were removed with the specimen, one of them was positive for disease on H&E, all the surgical margins were negative.

## 4. Discussion

Malignant melanomas are generally found in areas of skin exposed to the sun, but can also be present in nonexposed sites, such as genital tract and esophagus, among others [8]. 

Cervical melanoma arises from melanocytic cells of the cervix; in fact, the cervical epithelium is capable of forming the complete spectrum of melanocytic lesions, from benign lentigines to blue nevi to melanoma [10]. 

The usual form of presentation of primary melanoma of the cervix on physical examination is a polypoid exophytic mass, red, brown, grey, black, or blue in color [6], or a colorless in the case of amelanotic melanomas, which constitute up to 55% of cases at this anatomic site [12], presented with vaginal bleeding [1, 6, 8, 9]. Age range varies from 20 to 78 years [6], being more common between 60 and 70 years [1]. Due to that the cervix is an unusual site for this type of neoplasm, the International Federation of Gynecology and Obstetrics (FIGO) staging system for cervical cancer is used [11], rather than the Clark and Breslow scales, because the FIGO staging system correlates better with the prognosis.

Diagnosis of primary melanoma of the cervix entertains a high probability of being confused with another entity, due to the rarity of the disease [9]. Differential diagnosis between a primary cervical melanoma and a metastatic tumor is important because the latter can be part of a metastatic disease spreading to the cervix [1].

Norris and Taylor criteria are used [13] to distinguish whether it is a primary malignant melanoma of the cervix [6, 8, 12, 13]: (a) presence of melanin in the cervical epithelium; (b) absence of melanoma in another site of the body; (c) presence of binding activity in the cervical epithelium near the lesion; (d) if metastatic disease is found, it should be according to the cervical carcinoma pattern.

At the present moment there is no standard treatment for this disease, while there is no doubt that the surgical approach is the most usual and radical hysterectomy with or without pelvic lymphadectomy and/or superior vaginectomy is reported most frequently [1, 2, 7, 8], some authors entertain doubts concerning survival if pelvic lymphadectomy is performed [2]. Although there is no enough information about the real role of negative margins in primary melanoma of the cervix, the primary surgery should have the purpose of obtaining negative margins [6]; some authors recommend 2-cm margins as minimum [7, 9]. 

The role of radiotherapy (RT) has not been well established, but it has been demonstrated that RT reduces the tumor size [1, 8, 11]. The use of adjuvant pelvic RT is considered in the case of not obtaining a satisfactory surgical resection margins, when the parametrium is involved, or when lymph nodes are found to be involved [6]. Despite the low level of radiosensitivity exhibited by melanoma, the use of external or intracavitary RT is recommended after surgery, or palliatively when the tumor is inoperable because of its own conditions or those of the patient [1, 6, 8, 10].

No chemotherapy regimes have been reported that substantially may reduce the possibility of recurrence. Dacarbazin is utilized in advanced disease, and it has been observed that up to 20% of patients may have response [6, 7]. It has been proposed that the combination of cisplatin, bleomycin, and vinblastine can provoke a better response than the use of solely dacarbazine [1], while in other cases, the greater effectiveness of combining dacarbazine with vincristine and carmustine has been reported as well as immunotherapy utilizing local BCG or the transfusion of activated lymphocytes [10].

Average survival reported in the world literature of these patients ranges from 6 months to 14 years. The majority of them report that they succumb to the disease in the first 3 years after diagnosis [8]. Five-year survival after radical hysterectomy as only treatment is very low: less than 40% in stage I and 14% in stage II [1, 14]. 

The limited experience in the management of these cases, the unpredictable biological behavior of the disease, and the great variety of treatments employed render the choice of an optimal therapy for primary malignant melanoma of the cervix difficult [4, 7].

In our case, due to the magnitude of the tumor, it was decided to employ total pelvic exenteration, showing extraordinary results, because the patient continues to be alive and disease-free at 8 years of surgery, which is in contrast importantly with that reported in the literature after treatment with radical hysterectomy. We propose consideration of this surgical approach and adjuvant RT for primary melanoma of the cervix as the initial treatment of choice.

It is necessary to carry out long-term followup and biological research to obtain a consensus with regard to the diagnosis, treatment, and prognosis of the disease [11].

## 5. Conclusion

Primary malignant melanoma of the cervix is a rare disease with a poor prognosis, especially if it is not detected in a timely fashion or if it is not treated correctly. To date, no consensus has been established concerning treatment of primary melanoma of the cervix, but it is recommended that this be surgical, procuring the establishment of 2-cm margins, accompanied by radio- or chemotherapy. The majority of the case reports found suggest radical hysterectomy as the treatment indicated for these patients; notwithstanding this, survival is less than three years in general when managed in this manner.

Total pelvic exenteration for primary malignant melanoma of the cervix offers a feasible initial treatment for these unusual cases. It is necessary initiate collaborative studies and followup of patients to be able to achieve establishing a therapeutic approach that may offer the best results to these unfortunate patients.
